# EPG Recordings Reveal Differential Feeding Behaviors in *Sogatella furcifera* in Response to Plant Virus Infection and Transmission Success

**DOI:** 10.1038/srep30240

**Published:** 2016-08-05

**Authors:** Wenbin Lei, Pei Li, Yongqiang Han, Shaolong Gong, Lang Yang, Maolin Hou

**Affiliations:** 1 State Key Laboratory for Biology of Plant Diseases and Insect Pests, Institute of Plant Protection, Chinese Academy of Agricultural Sciences, Beijing 100193, China; 2 Southern Regional Collaborative Innovation Center for Grain and Oil Crops in China, Changsha 410128, China

## Abstract

Plant viruses are primarily transmitted by insect vectors and virus infection may influence on the vectors’ feeding behaviors. Using an electrical penetration graph, we detected that infection with the *Southern rice black-streaked dwarf virus* (SRBSDV) in the white-backed planthopper (WBPH) and in rice plants both altered the vector’s feeding behavior. When viruliferous WBPH (carrying SRBSDV) were fed on uninfected plants, they spent more time in salivation and phloem sap ingestion than non-viruliferous insects. In comparison with uninfected plants, infected plants showed an arrestant effect on non-viruliferous WBPH for phloem sap ingestion. Differential feeding behaviors were also detected between the WBPH that inoculated or acquired SRBSDV and those that failed to. The WBPH that inoculated SRBSDV exhibited more probing bouts, salivation events and phloem sap ingestion events and longer salivation than those that failed to. The WBPH that acquired SRBSDV were quicker to reach phloem and spent more time in phloem sap ingestion than those that failed to. These behavior alterations in the vector may have adaptive advantages for SRBSDV transmission and spread success because greater salivation by viruliferous vectors on uninfected hosts will promote virus inoculation, whereas more sap ingestion by non-viruliferous vectors on infected hosts will promote virus acquisition.

Pathogenic and parasite organisms may have interacted evolutionarily with their hosts and vectors to influence vectors’ performance and behavior favoring their spread^1^,^2^, which leads to the emergence of the “Host Manipulation Hypothesis” (HMH)^3^, the adaptive manipulation^4^ and behavioral manipulation^5^ hypotheses. Although documented mostly in animal pathosystems, such phenomena have only started to be recorded in plant pathosystems. In plant pathosystems, the plant is sessile, so the potential for behavioral manipulation is restricted to the vector, the mobile component in these systems^6^. The “Vector Manipulation Hypothesis” (VMH)^6^ was proposed to explain such evolutionary interactions in plant pathogens that enhance their spread to new hosts through their effects on the mobile vectors. Plant viruses are mostly transmitted by arthropods^7^ and are receiving much attention in studies of vector manipulation as a model plant pathosystem.

The *Southern rice black-streaked dwarf virus* (SRBSDV), a newly reported *Fijivirus* of the family Reoviridae^8^, has become increasingly prevalent in southern China, Vietnam and Japan^9^,^10^,^11^. SRBSDV is transmitted in a persistent, circulative and propagative manner^12^. Rice plants infected with SRBSDV present stunted and dark green leaves in the early stages and display small enations on the stems and abnormal tiller formation on the upper parts of the plants in the late stages^9^. The white-backed planthopper (WBPH), *Sogatella furcifera* (Hovath) (Hemiptera: Delphacidae), is the only confirmed vector of SRBSDV^13^. SRBSDV enters the midgut via the stylet, replicates in viroplasms and thereafter disseminates into the salivary gland for further inoculation. The WBPH vector can acquire SRBSDV after only 5 min of feeding on infected plants. At 24 h after virus ingestion, virus-positive rates of nymphs and adults reach 50.0% and 25.9%, respectively. The circulative transmission periods of SRBSDV in the WBPH range from 6 to 14 d, and the virus transmission rates of viruliferous nymphs and adults (carrying SRBSDV) are 54.3% and 42.9%, respectively^12^. SRBSDV cannot be transmitted by the brown planthopper, *Nilaparvata lugens* (Stål) (Hemiptera: Delphacidae), or the small brown planthopper, *Laodelphax striatellus* Fallen (Hemiptera: Delphacidae)^12^, which co-exist with the WBPH in paddy fields. Likewise, the virus cannot be transmitted via oviposition or seeds^9^.

In the interactions between plant-virus-vector, a plant virus can exert an influence on its vector’s developmental fitness. Like other persistent propagative viruses^14^, SRBSDV influences the performance of its vector. Our previous study demonstrated that the WBPH nymph duration as well as the female fecundity and longevity were significantly affected by SRBSDV, either directly (by infection of WBPH) or indirectly (by infection of rice plants)^15^. Other studies reported similar results for the same virus-vector interaction^13^,^16^.

A plant virus can also alter its vector’s behavior to influence the virus transmission and spread. One such behavior alteration concerns the vector’s orientation preference to virus-infected host plants. Wang *et al*.^17^ showed that SRBSDV-free WBPH were more attracted to SRBSDV-infected rice plants than to uninfected plants, whereas viruliferous WBPH preferred uninfected plants. Similar results were found in the interactions between the Luteovirus *Barley yellow dwarf virus* (BYDV) and its aphid vector, *Rhopalosiphum padi* L. (Hemiptera: Aphididae)^6^ and in the potato-*Myzus persicae* (Sulzer)-*Potato leaf roll virus* (PLRV) pathosystem^18^. Moreno-Delafuente *et al*.^19^ showed that the Geminivirus *Tomato yellow leaf curl virus* (TYLCV) induced an arrestant behavior in the whitefly *Bemisia tabaci* (Gennadius) (Hemiptera: Aleyrodidae) because viruliferous whitefly adults remained motionless for a longer time and moved more slowly than non-viruliferous whiteflies (not carrying the virus) after their first contact with eggplants, a non-host plant of this virus. Headspace volatiles from Luteovirus PLRV-infected mature leaves of *Solanum tuberosum* L. attracted the aphid *M. persicae* (Hemiptera: Aphididae)^20^. These results indicate that plant viruses might indirectly manipulate the orientation or settling behavior of their insect vectors, possibly through a virus-mediated plant volatile emission^21^,^22^. Such behavior manipulation may enhance the probability of virus transmission and spread.

Another manipulative behavior involves the vector’s probing and feeding behavior in response to its virus infection status. Male thrips *Frankliniella occidentalis* (Pergande) (Thysanoptera: Thripidae) infected with the Bunyavirus *Tomato spotted wilt virus* (TSWV) exhibited an increased feeding time and frequency of all feeding behaviors versus uninfected males^23^. Importantly, the same study found that infected males made almost three times more non-ingestion probes, which are a prerequisite for virus inoculation, compared with uninfected males^23^. Moreno-Delafuente *et al*.^19^ reported a more frequent feeding from phloem sieve elements, a larger number of phloem contacts and a longer duration of the salivation phase in phloem sieve elements in TYLCV-viruliferous *B. tabaci* than in non-viruliferous whiteflies. Alvarez *et al*.^20^ observed several differences in the plant penetration by *M. persicae* between PLRV-infected and uninfected potato plants but only after the infected plants showed visual symptoms of infection. More recently, Liu *et al*.^24^ recorded a more rapid probing and a higher number of feeding bouts by *B. tabaci* in TYLCV-infected tomato plants. These studies, based on detailed recordings of specific probing and feeding behaviors, imply that infection with a plant virus can directly (through the infected vectors) or indirectly (through infected host plants) alter the vector’s probing and feeding behavior in a way that increases the transmission efficiency of the virus.

Successful virus inoculation and acquisition is associated with the vector’s specific feeding behavior. For example, in the acquisition of BYDV by *R. padi*, the duration of the E waveform (phloem sap sucking) generated by aphids that successfully acquired BYDV was longer than in those that failed to ref. 25. Similarly, *Cicadulina spp*. that acquired the *Maize stripe virus* (MSV) successfully generated an L2 waveform (similar to the E waveform of aphids) more frequently^26^. A positive correlation was found between the Bromovirus *Cucumber mosaic virus* (CMV) acquisition by *Aphis gossypii* Glover (Hemiptera: Aphididae) and the generation of potential drop waveform (intracellular punctures)^27^. The successful inoculation of TYLCV by *B. tabaci* to tomato plants depends on the time duration spent in salivation into the phloem sieve elements^28^. These results demonstrate that the transmission of plant viruses is closely related to the specific feeding behaviors of the insect vectors. It is not clear if infection with SRBSDV will influence the vector’s feeding behaviors.

In this study, the probing and feeding behaviors of the WBPH in response to SRBSDV infection status and virus transmission success were recorded using an electrical penetration graph (EPG) technique. These EPG recordings were compared between viruliferous and non-viruliferous WBPH on uninfected rice seedlings, non-viruliferous WBPH on infected and uninfected rice seedlings, viruliferous WBPH that inoculated the virus to uninfected plants and those that failed to, and non-viruliferous WBPH that acquired the virus from infected plants and those that failed to. The transmission and spread of plant viruses depend on the feeding behavior of their vectors. Hence, according to the VMH, we hypothesize that SRBSDV can alter the behavior of its vector to enhance its transmission.

## Results

### Feeding behavior during virus inoculation access period (IAP)

**Figure 1:**
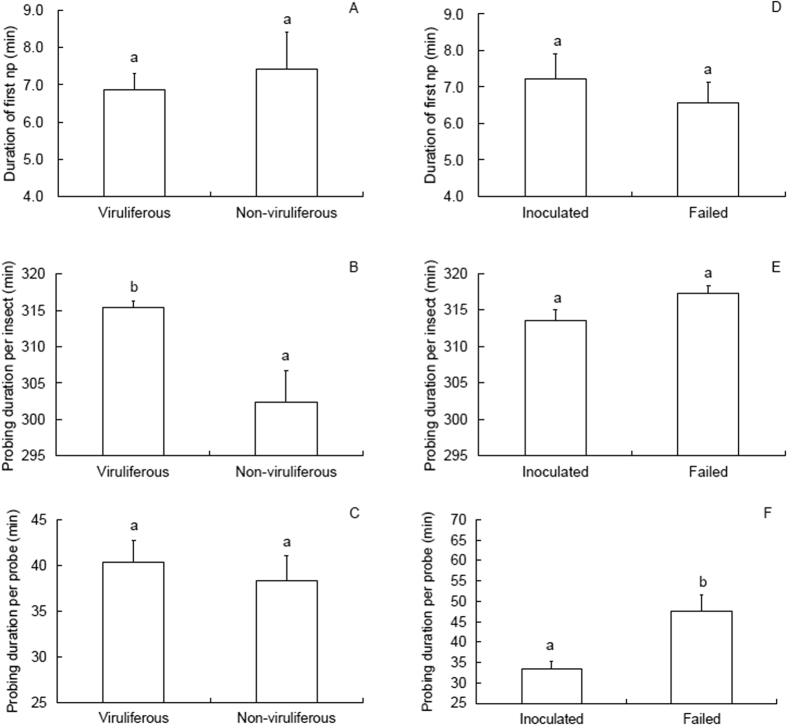
First np and probing duration (means ± SE) electrically recorded for 6 h with *Sogatella furcifera* macropterous females feeding on uninfected rice plants. (**A–C**) comparison between SRBSDV-viruliferous and non-viruliferous insects; (**D–F**) comparison between the insects that inoculated the virus and those that failed to.

The observed insects probed for more than 5 h (*PDI*, probing duration per insect) during the 6 h of the EPG recording period. When compared with the non-viruliferous WBPH, the viruliferous WBPH showed longer *PDI* on uninfected rice seedlings (Mann-Whitney *U*-test, *Z* = 2.438, *P* = 0.015; Fig. 1B). This pattern largely originated from the difference in the waveform duration per insect (*WDI*) of salivation and stylet movement (N2; *t* = 3.003, *P* = 0.003; Table 1) and phloem sap ingestion (N4-b; *t* = 2.23, *P* = 0.028; Table 1). The viruliferous insects also showed longer N2 waveform duration per event (*WDE*; *t* = 3.675, *P* < 0.001; Table 1) and a greater N4-b number of waveform events per insect (*NWEI*; *t* = 2.372, *P* = 0.025; Table 1) than their non-viruliferous counterparts.

Within the viruliferous WBPH, the insects that inoculated SRBSDV showed shorter probing duration per probe (*PDP*; *t* = 3.436, *P* = 0.001; Fig. 1F) and exhibited more events of penetration initiations (N1 *NWEI*; *t* = 3.412, *P* = 0.001; Table 2) than those that failed to. The N2 waveform represents the insect’s penetration of plant cells and salivation in the vascular bundle with its stylet. The WBPH that inoculated SRBSDV showed more N2 *NWEI* (*t* = 3.793, *P* < 0.001; Table 2) and longer N2 *WDI* (*t* = 3.725, *P* < 0.001; Table 2) than those that failed to. They were also characterized by more *NWEI* of stylet activities near (N3, *t* = 3.04, *P* = 0.003; Table 2) and in (N4-a, Mann-Whitney *U*-test, *Z* = 3.224, *P* = 0.001; Table 2) the phloem region and shorter *WDE* in N4-a (*t* = 4.274, *P* < 0.001; Table 2) than the insects that failed in inoculation.

### Feeding behavior during virus acquisition acess period (AAP)

**Figure 2:**
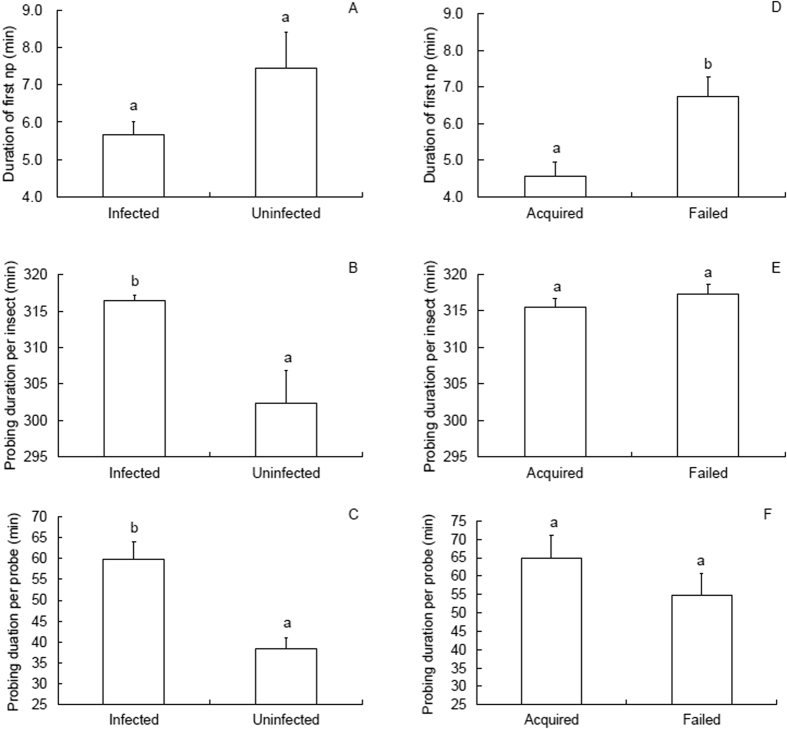
First np and probing duration (means ± SE) electrically recorded for 6 h with non-viruliferous *Sogatella furcifera* macropterous females feeding on infected or uninfected rice plants. (**A–C**) comparison between insects feeding on infected and uninfected plants; (**D–F**) comparison between the insects that acquired SRBSDV and those that failed to.

During virus AAP, the non-viruliferous WBPH exhibited longer *PDI* (Mann-Whitney *U*-test, *Z* = 2.697, *P* = 0.007; Fig. 2B) and *PDP* (Mann-Whitney *U*-test, *Z* = 3.704, *P* < 0.001; Fig. 2C) on SRBSDV-infected than on uninfected rice seedlings. Most strikingly, a similar situation occurred for the N4-b waveform, i.e., the phloem sap ingestion phase. Insects feeding on infected rice seedlings spent more time ingesting phloem sap (N4-b) than those feeding on uninfected plants, as indicated by both the *WDE* (Mann-Whitney *U*-test, *Z* = 3.955, *P* < 0.001; Table 3) and the *WDI* (*t* = 5.974, *P* < 0.001; Table 3). In contrast, for the extracellular stylet activity near the phloem region (N3), insects feeding on infected plants exhibited a significantly shorter *WDI* than those feeding on uninfected plants (Mann-Whitney *U*-test, *Z* = 4.373, *P* < 0.001; Table 3). Furthermore, significant differences were found in the *NWEI* of N1, N2, N3 and N4-a, which were fewer for the WBPH feeding on infected than on uninfected rice seedlings (*t* > 3.023, *P* < 0.003; Table 3).

In comparison to the virus-not-acquired WBPH feeding on infected rice plants, the virus-acquired insects showed shorter duration of first np per insect (*DFnp*; *t* = 3.467, *P* = 0.001; Fig. 2D). The virus-acquired insects also showed shorter *WDI* in both N2 (Mann-Whitney *U*-test, *Z* = 3.447, *P* = 0.001) and N4-a (Mann-Whitney *U*-test, *Z* = 6.658, *P* < 0.001). Likewise, the *WDE* in N4-a was shorter in virus-acquired insects (*t* = 5.569, *P* < 0.001; Table 4), whereas these insects exhibited longer *WDE* (*t* = 3.569, *P* = 0.001) and *WDI* (Mann-Whitney *U*-test, *Z* = 6.366, *P* < 0.001; Table 4) in N4-b than the virus-not-acquired insects. Furthermore, the insects that succeeded in virus acquisition were characterized by fewer *NWEI* of N2, N3 and N4-a (*t* > 2.422, *P* < 0.018; Table 4).

## Discussion

Previous reports show that the transmission of plant viruses is closely linked to the feeding behavior of sucking insect vectors. Hence, monitoring the feeding process can reveal the behavioral mechanism of plant virus transmission by insect vectors^19^,^23^,^25^,^28^,^29^,^30^,^31^. In the present study, EPG recordings revealed a differential probing and feeding behavior in the WBPH vector in response to the SRBSDV infection status and transmission success, indicating a manipulation of vector behavior by the virus.

When viruliferous and non-viruliferous *S. furcifera* were compared on uninfected rice plants, the viruliferous insects spent more time in salivation (N2) and phloem sap ingestion (N4-b) compared with non-viruliferous insects. Similar results have been reported in the thrips *F. occidentalis*, where TSWV-infected male thrips spent more time feeding than uninfected males^23^, and in the whiteflies *B. tabaci*, where TYLCV-viruliferous insects sustained a longer duration of the salivation phase in phloem sieve elements than non-viruliferous whiteflies^19^. An early study by Martín *et al*.^32^ on C*ucumber mosaic virus* (CMV) and *Potyvirus potato virus Y* (PVY), which are transmitted by both *Aphis gossypii* Glover and *Myzus persicae* Sulzer, showed that the transmission of plant viruses from viruliferous insect vectors to uninfected plants was tightly related to the production of II-1 waveforms (salivation of insects). In these studies, the plants are uninfected; the differences in probing behavior between the viruliferous and non-viruliferous vectors can result from the virus infection status of the vector, i.e. the viruses have directly altered the probing behavior of their vectors. These behavioral alterations may promote virus spread, since a long salivation duration preceding the phloem sap ingestion by the viruliferous vectors can help inoculate the viruses to uninfected plants.

When the non-viruliferous WBPH exposed to SRBSDV-infected plants were compared with those exposed to uninfected rice plants, the insects on infected plants were quicker to probe (a shorter *DFnp*) and probed longer (both *PDI* and *PDE*; Table 3), spent more time (both *WDE* and *WDI*) in phloem sap ingestion (N4-b) and exhibited fewer probing events (*NWEI*, with the exception of N4-b; Table 4). These results clearly show that infected rice plants have an arrestant effect on non-viruliferous WBPH for probing, especially for phloem sap ingestion. Our results are consistent with those reported by Liu *et al*.^24^ in that they also recorded a quicker probing by the *B. tabaci* whitefly on TYLCV-infected tomato plants, and in aphid vectors feeding on BYDV-infected versus uninfected oats^33^ and in aphids *M. persicae* feeding on PLRV-infected versus uninfected potato plants^20^. The quicker probing recorded in these studies confirm that non-viruliferous vectors prefer virus-infected plants over uninfected plants^6^,^17^,^18^. These findings points to the possibility that virus infection has mediated alterations of host plant’s secondary chemistry^21^, such as enhanced emission of volatile organic compounds^22^. Our and others’ results indicate that virus infection of host plants can indirectly change the feeding behavior of vectors in favor of virus spread, because the quicker probing and the extended phloem sap ingestion on the infected host plants by non-viruliferous insects can enhance the chances of virus acquisition.

In contrast to the WBPH that failed to inoculate SRBSDV, those that succeeded in inoculation showed a shorter probing duration per probe (*PDP*; Table 1), more probing attempts (N1 *NWEI*), more salivation (N2) waveform number and longer N2 waveform duration and greater stylet activity near and in the phloem region (N3 and N4-a *NWEI*; Table 2). The N1 waveform correlates with the piercing behavior; therefore, more piercing behavior will increase the possibility of successful virus inoculation. Most plant viruses were inoculated via intracellular salivation by viruliferous vectors^34^. The increased number and extended duration of salivation (N2) events per insect in the viruliferous insects that successfully inoculated SRBSDV can be conducive to successful virus inoculation. Our results are consistent with several previous reports. The salivation duration of BYDV-viruliferous *R. padi* was longer when the aphids transmitted the virus to uninfected barley plants than when they did not^25^. The transmission rate of TYLCV by viruliferous *B. tabaci* whiteflies to uninfected tomato plants was higher in insects that salivated into the phloem sap than those that did not^28^. Additionally, these two studies also recorded a significant increase in the bouts of phloem salivation in insects that succeeded in inoculation of the viruses^25^,^28^. Despite these similar reports, Stafford *et al*.^35^ showed that inoculation with the Geminivirus *Beet severe curly top virus* (BSCTV) by the beet leafhopper, *Circulifer tenellus*, depended only on the production of the D1 waveform (salivation) instead of on the duration of the D1 waveform. The difference may lie in the use of a different strategy by leafhoppers than by aphids and whiteflies for phloem ingestion; the phloem sap ingestion by the leafhopper is not necessarily preceded by salivation into the phloem^36^ as it is for aphids and whiteflies. This same mechanism may also stand true for the difference between the WBPH and the leafhopper. Greater stylet activity near or in the phloem region in the WBPH that inoculated SRBSDV than in those that failed in inoculation indicates that these activities may increase the virus inoculation. Therefore, our results indicate that the successful inoculation of the SRBSDV by WBPH relies on extended salivation duration and frequent stylet activities before phloem sap ingestion.

Interestingly, the WBPH that acquired SRBSDV settled on rice plants without piercing activities for a shorter time (a shorter *DFnp*, Table 3) before the first probe compared with those that failed to. They generated fewer waveforms of salivation (N2) and stylet activities near (N3) and in (N4-a) the phloem region than the insects that failed in virus acquisition (Table 4). They also spent less total time (*WDI*) in salivation (N2) and less event time (*WDE*) and total time (*WDI*) in stylet activities in the phloem region (N4-a) but more event time and total time in phloem sap ingestion (N4-b) than the WBPH that failed to acquire the virus (Table 4). These results show that the WBPH that were successful in virus acquisition ‘waste’ less time before the phloem sap ingestion event but instead spend more time on the ingestion itself. The phloem sap of SRBSDV-infected rice plants contains the virus particles^9^. A longer phloem sap ingestion by WBPH on infected rice plants can increase the possibility of virus acquisition. Similar results were reported for *R. padi*, where non-viruliferous *R. padi* acquired BYDV at a higher rate on BYDV-infected wheat plants when the aphids generated more E2 waveforms (phloem sap ingestion)^25^. From these results, it can be concluded that the successful acquisition of the SRBSDV by WBPH depends on extended phloem sap ingestion.

Overall, our results indicate that the virus infection status of both the vector and the host plant alters the vector’s probing and feeding behavior and that a successful virus transmission is characterized by specifc probing and feeding behavior, offering quantitative evidence for the VMH. These behavior alterations have adaptive advantages for the virus transmission and spread, i.e., a long salivation duration preceding the phloem sap ingestion by the viruliferous vector can help inoculate the virus to uninfected plants, and the extended phloem sap ingestion from the SRBSDV-infected rice plants by non-viruliferous insects can enhance the chances of virus acquisition. These findings further our understanding of the evolution of host-vector interactions and the vector behavioral mechanisms for plant virus transmission and spread.

## Methods

### Test insects and plants

A sample of SRBSDV-infected rice seedlings was kindly provided by Dr. Zhou (South China Agricultural University). The virus stock culture was maintained using WBPH as vector and three-leaf to tillering stage rice seedlings (Taichung Native 1, TN1) as host plants within 80 mesh insect-proof cages (50 × 50 × 50 cm) in a climate chamber at 27 ± 1 °C, a relative humidity (RH) of 75–80% and a photoperiod of 16L:8D. Stock cultures of WBPH exposed and not exposed to SRBSDV were also maintained under the same conditions in separate insect-proof mesh cages.

Macropterous WBPH females emerged within 24 h from the stock cultures were used as the test insects in the EPG recordings. To obtain the females, 5^th^ instar nymphs were transferred from a stock culture into glass tubes (2.5 × 15 cm) each with a rice seedling and 10 ml of Kimura solution. Newly emerged macropterous WBPH females were transferred to a new glass tube with a rice seedling within a 12 h period and then used in the experiments.

Potted rice seedlings (TN1) cultured in plastic pots (5 cm diameter × 7 cm height) were used in the EPG recordings. To obtain infected rice seedlings, the virus-free seedlings (approximately 20 d old) were individually confined with 3 viruliferous 4^th^ instar WBPH nymphs for 24 h. Ten days after the exposure, a leaf was cut from each of the seedlings and tested for the SRBSDV infection status using RT-PCR (SRBSDV positive or not). The infected and uninfected rice plants were maintained separately in insect-proof cages inside climate chambers.

### Detection of the SRBSDV infection status by RT-PCR

The SRBSDV-infection status of rice seedlings and WBPH were detected by one-step RT-PCR^37^. Briefly, the total RNA of rice or WBPH was extracted according to the methods provided by the manufacture of TRIzol reagent (Takara Biotechnology Co. Ltd., Dalian, China). RNA was then reverse transcribed and amplified using the primer S10-oF/S10-oR(5′-CGCGTCATCTCAAACTACAG-3′/5′-TTTGTCAGCATCTAAAGCGC-3′). The rice seedlings and the WBPH were regarded as either infected or viruliferous if a 682 bp positive band was present.

### Treatment groups and EPG recordings

The feeding behavior of the WBPH during the SRBSDV inoculation access period (IAP) and acquisition access period (AAP) were recorded using a Giga-8 direct current electrical penetration graph (DC-EPG) amplifier with a 10^9^-Ω input resistance in a Faraday cage (manufactured by Wageningen University, Wageningen, The Netherlands). For the IAP, viruliferous WBPH feeding on uninfected rice seedlings were electrically monitored to obtain an EPG; and for the AAP, non-viruliferous WBPH feeding on infected rice seedlings were monitored. A control, i.e., non-viruliferous WBPH feeding on uninfected rice seedlings, was included in the EPG recordings.

The obtained macropterous WBPH females were starved for 2 h (supplied with water only on filter paper in a flask) before the EPG recordings. To facilitate the attachment of thin gold wires to the planthoppers, each WBPH female was immobilized using a micro-vacuum pump (QC-1S, Beijing Municipal Institute of Labour Protection) by suctioning on its abdomen. Then, one end of a gold wire (18 μm diameter × 3–4 cm length) was attached to the dorsal thorax of the insect using a drop of water-soluble silver glue and allowed to dry for approximately 10 s. The wired insect was then connected to the amplifier through a copper nail that was inserted into the EPG probe and placed on a rice leaf sheath of a potted plant. This treatment did not hamper the feeding of the planthopper. A copper wire (2 mm diameter × 10 cm length) connected to the amplifier and vertically inserted into the pot soil was used as the plant electrode. The EPG signals were digitized with a converter (DI710-UL, Dataq, Akron, USA), and the gain of the amplifier was acquired and stored with the PROBE 3.4 software (Wageningen University, Wageningen, The Netherlands). The substrate voltage was adjusted so that the EPG signals fit into the +5 V to −5 V window provided by the PROBE software. A 1 h acclimation period was set between the wiring time and the beginning of the EPG recording as described by Moreno-Delafuente *et al*.^12^. Each insect was continuously recorded for 6 h. A different WBPH and plant was used for each recording. The EPG recordings were conducted in a quiet room with ambient conditions of 27 ± 2 °C and RH 70 ± 5%. Immediately after each EPG recording in the IAP, the virus infection status of the test insects was individually assessed by RT-PCR. WBPH that showed positive RT-PCR results were considered as valid samples and included in the final analysis. After 10 d of the EPG recordings, rice seedlings in the IAP and planthoppers in the AAP were individually tested to assess the SRBSDV infection status. From these test results, the WBPH in IAP were classified as either viruliferous WBPH that inoculated SRBSDV or those that failed to. In addition, the WBPH in AAP were divided into either non-viruliferous WBPH that acquired SRBSDV or those that failed to. Therefore, a total of five groups of WBPH were differentiated. For each of the five groups, a total of 34 valid repetitions were obtained.

### EPG waveforms and parameters

Similar to previous reports^38^,^39^, six distinctive waveforms were observed during probing by the white-backed planthopper in this study. These waveforms were interpreted with reference to the studies by Khan and Saxena^38^ and Seo *et al*.^39^, i.e., non-penetration (np), penetration initiation (N1), salivation and stylet movement (N2), extracellular stylet activity near the phloem region (N3), intracellular stylet activity in the phloem region without ingestion (N4-a) and phloem sap ingestion (N4-b).

The EPG parameters were calculated for each recording with reference to Sarria *et al*.^40^. A probe was defined to start from the insect stylet insertion to the stylet withdrawal^23^. Referring to Moreno-Delafuente *et al*.^19^ and Backus *et al*.^41^, six parameters were obtained from the EPG recordings: duration of first np per insect (*DFnp*, min); probing duration per insect (*PDI*, min), which is the sum of the durations of each event of all the waveforms made by an insect; probing duration per probe (*PDP*, min), which is the sum of the probing duration of an insect divided by the number of probes made by the insect; number of waveform events per insect (*NWEI*), which is the sum of the number of events of a particular waveform; waveform duration per event (*WDE*, min), which is the sum of the durations of each event for a particular waveform divided by the number of events of that particular waveform; and waveform duration per insect (*WDI*, min), which is the sum of the durations of each event of all the waveforms made by an insect. All the parameters were averaged across the number of insects, probes or events (in the sense of Backus *et al*.^41^).

### Statistical analysis

The EPG recording data were normalized with a square root (X + 1) transformation^42^. A comparison of the parameters was performed between viruliferous and non-viruliferous WBPH feeding on uninfected plants as well as between viruliferous WBPH that inoculated SRBSDV and those that failed to. Another comparison was conducted between non-viruliferous WBPH feeding on infected and uninfected rice seedlings as well as between non-viruliferous WBPH that acquired SRBSDV and those that failed to. The transformed data that followed a Gaussian distribution were compared with a Student t-test. When the assumption of equal variance was not met, the non-parametric Mann-Whitney *U*-test was used for comparison. These statistical analyses were performed using SPSS software^43^.

## Additional Information

**How to cite this article**: Lei, W. *et al*. EPG Recordings Reveal Differential Feeding Behaviors in *Sogatella furcifera* in Response to Plant Virus Infection and Transmission Success. *Sci. Rep.* **6**, 30240; doi: 10.1038/srep30240 (2016).

## Figures and Tables

**Table 1:** Electrically recorded feeding behavior of SRBSDV-viruliferous or non-viruliferous *Sogatella furcifera* macropterous females on uninfected rice plants.

Waveforms	WBPH	*NWEI*	*WDE* (min)	*WDI* (min)
N1	Non-viruliferous	9.0 ± 0.55 a	0.26 ± 0.03 a	2.36 ± 0.28 a
	Viruliferous	9.2 ± 0.45 a	0.28 ± 0.02 a	2.59 ± 0.24 a
N2	Non-viruliferous	8.2 ± 0.53 a	1.47 ± 0.18 a	12.25 ± 1.57 a
	Viruliferous	8.2 ± 0.41 a	2.29 ± 0.14 b	17.91 ± 1.17 b
N3	Non-viruliferous	7.0 ± 0.45 a	9.80 ± 1.03 a	59.79 ± 4.41 a
	Viruliferous	6.6 ± 0.27 a	9.09 ± 0.39 a	54.81 ± 1.49 a
N4-a	Non-viruliferous	5.2 ± 0.30 a	21.42 ± 2.22 a	94.77 ± 5.12 a
	Viruliferous	5.6 ± 0.24 a	18.48 ± 0.97 a	92.55 ± 3.34 a
N4-b	Non-viruliferous	3.5 ± 0.22 a	41.19 ± 2.36 a	133.26 ± 6.36 a
	Viruliferous	4.2 ± 0.18 b	39.93 ± 2.05 a	147.53 ± 3.68 b

Data are expressed as the means ± SE. The means of a waveform parameter followed by the same letters were not significantly different according to a Student t-test for Gaussian distributed variables and a Mann Whitney *U*-test for non-Gaussian distributed variables. *NWEI*, number of waveform events per insect; *WDE*, waveform duration per event; and *WDI*, waveform duration per insect. These abbreviations are same for the other tables.

**Table 2:** Electrically recorded feeding behavior of SRBSDV-viruliferous *Sogatella furcifera* macropterous females on uninfected rice plants: comparison between the insects that inoculated SRBSDV and those that failed to.

Waveforms	WBPH	*NWEI*	*WDE* (min)	*WDI* (min)
N1	Inoculated	10.6 ± 0.67 b	0.27 ± 0.04 a	2.99 ± 0.43 a
	Failed	7.8 ± 0.49 a	0.28 ± 0.02 a	2.19 ± 0.22 a
N2	Inoculated	9.6 ± 0.63 b	2.53 ± 0.22 a	22.20 ± 1.76 b
	Failed	6.8 ± 0.40 a	2.05 ± 0.17 a	13.61 ± 1.17 a
N3	Inoculated	7.4 ± 0.39 b	8.44 ± 0.53 a	56.84 ± 2.25 a
	Failed	5.8 ± 0.32 a	9.75 ± 0.54 a	52.78 ± 1.94 a
N4-a	Inoculated	6.4 ± 0.35 b	14.76 ± 0.98 a	87.02 ± 4.24 a
	Failed	4.8 ± 0.26 a	22.21 ± 1.44 b	98.07 ± 5.03 a
N4-b	Inoculated	4.4 ± 0.30 a	37.82 ± 2.92 a	144.48 ± 5.02 a
	Failed	3.9 ± 0.19 a	42.04 ± 2.88 a	150.59 ± 5.42 a

**Table 3:** Electrically recorded feeding behavior of SRBSDV- non-viruliferous *Sogatella furcifera* macropterous females on infected or uninfected rice plants.

Waveforms	Rice plant	*NWEI*	*WDE* (min)	*WDI* (min)
N1	Uninfected	9.0 ± 0.55 b	0.26 ± 0.03 a	2.36 ± 0.28 a
	Infected	6.8 ± 0.39 a	0.28 ± 0.01 a	1.77 ± 0.12 a
N2	Uninfected	8.2 ± 0.53 b	1.47 ± 0.18 a	12.25 ± 1.57 a
	Infected	5.7 ± 0.29 a	1.74 ± 0.16 a	8.78 ± 0.67 a
N3	Uninfected	7.0 ± 0.45 b	9.80 ± 1.03 a	59.79 ± 4.41 b
	Infected	4.9 ± 0.23 a	9.19 ± 0.51 a	40.26 ± 1.50 a
N4-a	Uninfected	5.2 ± 0.30 b	21.42 ± 2.22 a	94.77 ± 5.12 a
	Infected	4.2 ± 0.19 a	20.99 ± 0.93 a	83.03 ± 4.02 a
N4-b	Uninfected	3.5 ± 0.22 a	41.19 ± 2.36 a	133.26 ± 6.36 a
	Infected	3.3 ± 0.12 a	61.83 ± 3.18 b	182.52 ± 4.82 b

**Table 4:** Electrically recorded feeding behavior of non-viruliferous *Sogatella furcifera* macropterous females on infected rice plants: comparison between the insects that acquired SRBSDV and those that failed to.

Waveforms	WBPH	*NWEI*	*WDE* (min)	*WDI* (min)
N1	Acquired	6.1 ± 0.45 a	0.28 ± 0.02 a	1.64 ± 0.17 a
	Failed	7.5 ± 0.61 a	0.27 ± 0.02 a	1.90 ± 0.18 a
N2	Acquired	5.0 ± 0.33 a	1.44 ± 0.13 a	6.62 ± 0.42 a
	Failed	6.4 ± 0.45 b	2.05 ± 0.28 a	10.94 ± 1.17 b
N3	Acquired	4.3 ± 0.25 a	9.80 ± 0.62 a	38.10 ± 1.18 a
	Failed	5.5 ± 0.36 b	8.58 ± 0.80 a	42.41 ± 2.73 a
N4-a	Acquired	3.7 ± 0.19 a	16.81 ± 1.05 a	57.03 ± 1.76 a
	Failed	4.7 ± 0.30 b	25.16 ± 1.15 b	109.03 ± 4.63 b
N4-b	Acquired	3.2 ± 0.15 a	71.65 ± 4.07 b	212.06 ± 2.17 b
	Failed	3.4 ± 0.19 a	52.01 ± 4.31 a	152.98 ± 6.07 a
